# Supplementary material for: Efficacy Validation of SARS-CoV-2-Inactivation and Viral Genome Stability in Saliva by a Guanidine Hydrochloride and Surfactant-Based Virus Lysis/Transport Buffer
Source: Viruses. 2023 Feb 11;15(2):509. doi: 10.3390/v15020509 (PMC9959814; doi:10.3390/v15020509)
Supplement: Supplementary file 1 [file viruses-15-00509-s001.zip › viruses-2146135-supplementary.pdf]

Supplementary materials

Table S1. Viral gene positivity ratio in SARS-CoV-2-spiked saliva samples with different viral titers for determination of the viral titer LOD

**Viral titer in saliva samples (log_{10} $TCID_{50}$/mL)**	**Replicates (Positive/total)**	**% Positive**
3	4/4	100%
1	4/4	100%
0	3/4	75%
−0.3	4/4	100%
−1	1/4	25%
−1.3	0/4	0%
−2	0/4	0%

Table S2. Viral gene positivity ratio of 2-fold serial dilutions of Takara SARS-CoV-2 Positive Control for determination of the viral gene copy LOD

**Viral gene copy no. (copy/μL)**	**Replicates (Positive/total)**	**% Positive**
100	4/4	100%
50	4/4	100%
25	2/4	50%
12.5	1/4	25%
6.25	1/4	25%
3.125	0/4	0%
1.625	0/4	0%

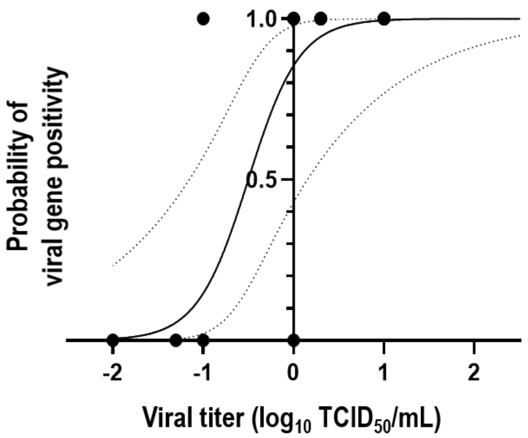

Figure S1. A simple logistic regression curve for the viral titer LOD determination related to Table S1. Dotted curves indicate the 95% asymptomatic confidence interval bands.

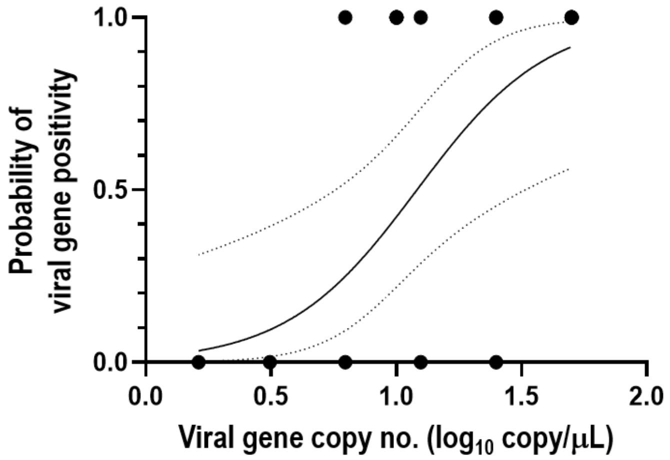

Figure S2. A simple logistic regression curve for the viral gene copy LOD determination related to Table S2. Dotted curves indicate the 95% asymptomatic confidence interval bands.

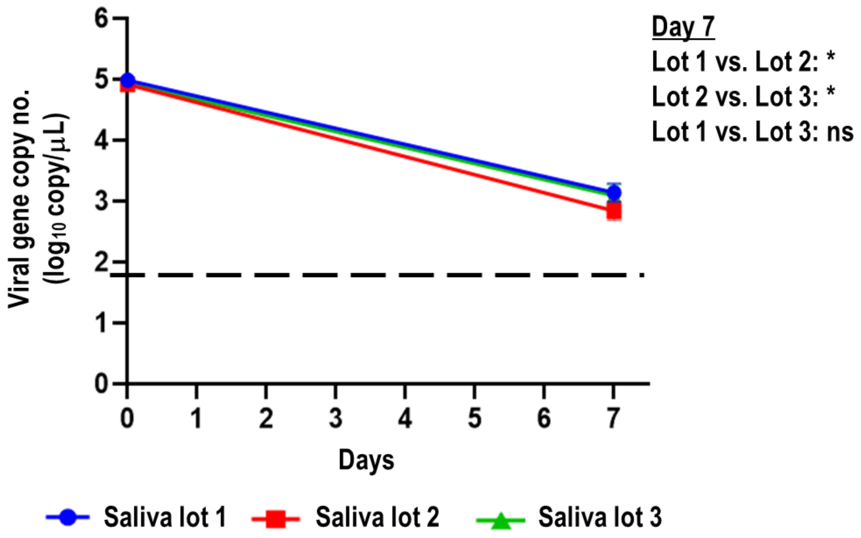

Figure S3. Effect of the difference of saliva lots on viral RNA stability in Prep Buffer A. Three different lots of SARS-CoV-2-spiked saliva (lots 1–3) with a high viral titer (3 $\log_{10}$ $TCID_{50}$/mL) were treated with Prep Buffer A and stored at 35°C for 7 days. The Kruskal–Wallis test with Dun's multiple comparison test was performed to analyze the statistical significance in viral gene copy numbers at day 7 among the saliva lots (n = 8 per group; *$p < 0.05$; ns: not significant). The black dashed line indicates the viral gene copy LOD (1.845 $\log_{10}$ copies/μL).

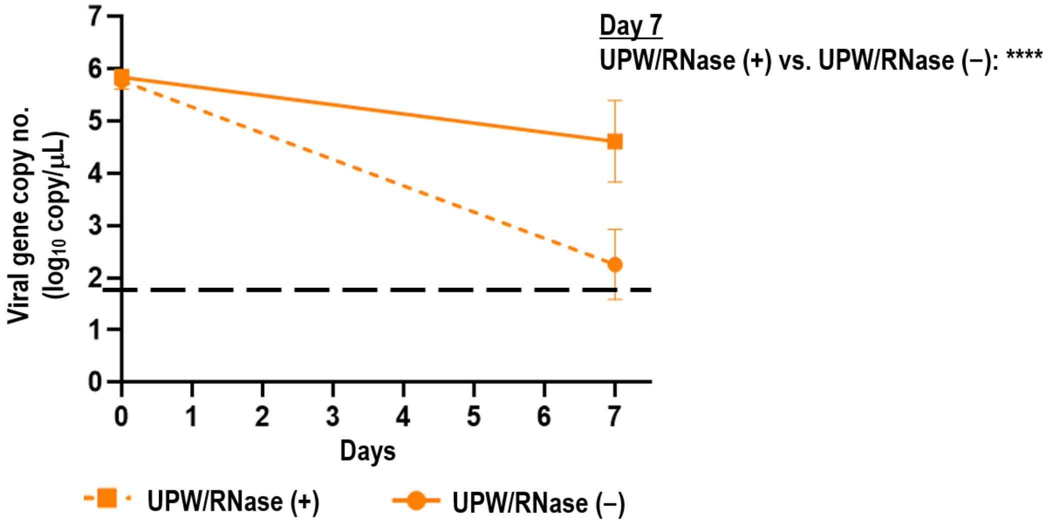

Figure S4. Direct impact of UPW on SARS-CoV-2 RNA. Viral RNA extracted from SARS-CoV-2 solution was treated with UPW and stored at 35°C for 7 days in the presence or absence of RNase. The unpaired *t*-test was performed to analyze the statistical difference in viral gene copy numbers at day 7 between the UPW/RNase (+) and UPW/RNase (−) groups (n = 8 per group; ****$p < 0.0001$). The black dashed line indicates the viral gene copy LOD (1.845 $\log_{10}$ copies/μL).
